# Comparative evaluation of lateral flow assays to diagnose chronic *Trypanosoma cruzi* infection in Bolivia

**DOI:** 10.1371/journal.pntd.0012016

**Published:** 2024-03-04

**Authors:** Ronald López, Andrea García, José Jorge Chura Aruni, Victor Balboa, Andrea Rodríguez, Berra Erkosar, Aurélie Kamoun, Marcelo Rodriguez, Evelin Fortun, Laura C. Bohorquez

**Affiliations:** 1 Instituto Nacional de Laboratorios de Salud (INLASA), La Paz, Bolivia; 2 FIND, Campus Biotech, Chemin des Mines 9, Geneva, Switzerland; Universidade Federal de Santa Catarina, BRAZIL

## Abstract

Bolivia has the highest incidence of Chagas disease (CD) worldwide. Caused by the parasite *Trypanasoma cruzi*, CD is generally a chronic condition. Diagnosis is logistically and financially challenging, requiring at least two different laboratory-based serological tests. Many CD cases are missed; in Bolivia it is estimated just 6% of individuals chronically infected with *T*. *cruzi* get diagnosed. Achieving control on the way to elimination of CD requires a radical simplification of the current CD testing pathways, to overcome the barriers to accessing CD treatment. We aimed to generate unbiased performance data of lateral flow assays (LFAs) for *T*. *cruzi* infection in Bolivia, to evaluate their usefulness for improving *T*. *cruzi* diagnosis rates in a precise and efficient manner.

This retrospective, laboratory-based, diagnostic evaluation study sought to estimate the sensitivity/specificity of 10 commercially available LFAs for *T*. *cruzi*, using the current CD diagnostic algorithm employed in Bolivia as the reference test method. All tests were blinded at the study site and performed by three operators. In total, 470 serum samples were tested, including 221 and 249 characterized as CD-positive/-negative, respectively. The LFAs were scored according to their relative importance using a decision-tree-based algorithm, with the mean decrease in Gini index as the scoring metric.

The estimates of sensitivities ranged from 62.2–97.7% (95% confidence interval (CI) lower bound 55.0–94.7%); for specificities the range was 78.6–100% (95% CI lower bound 72.0–97.5%); 5/10 and 6/10 tests had sensitivity >90% and specificity >95%, respectively. Four LFAs showed high values of both sensitivity (93–95%) and specificity (97–99%). The agreement between 6 LFAs and the reference tests was almost perfect (Kappa 0.83–0.94). Most LFAs evaluated thus showed performances comparable with current laboratory-based diagnostic methods.

## Author summary

Chagas disease (CD) is caused by the parasite *Trypanasoma cruzi*. CD is generally a chronic condition. Individuals living in vulnerable situations and marginalized populations are often more susceptible to CD infection and morbidity, determined by, among other factors, a lack of access to timely diagnosis and care. However, diagnosing CD is difficult and expensive, requiring at least two different laboratory-based tests in highly specialized facilities, and many cases of CD go undiagnosed. In Bolivia, which has the highest incidence of CD worldwide, it is estimated that just 6% of chronically infected individuals get diagnosed. Lateral flow assays (LFAs) are tests that could be used to improve case management, especially in hard-to-reach populations, as they do not need specialized laboratories or highly trained people to use them. We carried out a study to evaluate LFAs that can be used for the rapid detection of CD infection, to evaluate their usefulness for diagnosis. Most of the tests we evaluated under controlled laboratory conditions were comparable to those of current laboratory-based diagnostic methods.

## Introduction

Also known as American trypanosomiasis, Chagas disease (CD) is caused by the parasite *Trypanosoma cruzi* (*T*. *cruzi*). It is endemic in 21 Latin American countries, with around 6 million people infected; due to the migration of infected individuals, 70 million people worldwide are at risk of *T*. *cruzi* infection. Bolivia has the highest incidence of CD globally and is ranked fourth highest in terms of CD prevalence relative to its population, exceeded only by Argentina, Brazil, and Mexico [1]. It is estimated that more than 600,000 people in Bolivia are infected with *T*. *cruzi*, and 500,000 people are at risk of infection; 8,000 new cases occur annually due to vectorial transmission, while there are around 600 cases due to congenital transmission [2]. In 2015, a total of 30,454 individuals were diagnosed with CD, but just 10% of them started treatment [2,3]. Clearly, many cases of CD go undiagnosed; in Bolivia, it is estimated just 6% of individuals chronically infected with *T*. *cruzi* get diagnosed, limiting people’s access to timely healthcare.

CD is predominately a vector-borne disease, with *T*. *cruzi* parasites transmitted by contact with the feces or urine of infected blood-sucking triatomine bugs. The parasites can also be transmitted via contaminated food, blood transfusion, organ transplantation, laboratory accidents, and, importantly, via maternal transmission. CD presents in two phases: acute and chronic. The acute phase lasts for around two months following infection. During the chronic phase, the parasites remain hidden, mainly in the heart and digestive muscles. In subsequent years, the infection can lead to sudden death due to cardiac arrhythmias or progressive heart failure [4]. CD can be treated with benznidazole and nifurtimox. However, their efficacy diminishes the longer an individual has been infected, and adverse reactions become more frequent with increasing age.

In recent years there has been a substantial reduction in CD transmission due to a variety of factors, including multinational activities for the control of vectorial and transfusion-based transmission, prompt treatment of cases, and improved hygiene and food safety [1]. In Bolivia, great efforts have been made to tackle CD, especially in terms of vector control; however, CD remains a chronic condition and poses a long-term challenge for the prevention and control of non-vectorial transmission.

According to Bolivian and international guidelines [5,6], the current diagnostic algorithm recommended for patients with suspected chronic *T*. *cruzi* infection is the agreement of two laboratory-based serological tests with antigens that detect different antibodies against *T*. *cruzi*, such as an enzyme-linked immunosorbent assay (ELISA), indirect hemagglutination inhibition (HAI) assay, or indirect immunofluorescence (IIF) assay, plus a third serological test in the case of discordant results. In areas of high endemicity of *T*. *cruzi* in Bolivia, where there is often limited access to laboratory-based tools, blood samples are collected from pregnant women at their first antenatal care visit to screen them for *T*. *cruzi* infection. These samples are first tested using a lateral flow assay (LFA); this is followed by confirmation at the nearest laboratory, using a serological test, plus a third serological test if there are conflicting results.

ELISAs and LFAs are recommended for population-level studies into the prevalence of CD, while a modified, highly sensitive type of ELISA, the chemiluminescent microparticle immunoassay (CMIA), is recommended when screening for CD in hemotherapy services. The ELISA and chemiluminescent immunoassays (CLIAs) can detect a variety of antibodies against *T*. *cruzi*, with good analytical performance, but require a laboratory, specialized personnel, and many hours to obtain the results. LFAs are rapid diagnostic tests (RDTs) for antibody detection, delivering results in minutes, but there is a lack of independent performance data for these tests. Although there are at least 14 LFAs registered for use in endemic countries, including Bolivia, that can be used to detect *T*. *cruzi* infection, they are currently not widely used in public health systems.

More than 6,000 strains of *T*. *cruzi* have been identified; these have been classified into seven discrete typing units (DTUs), six referred to as TcI to TcVI, along with a seventh, Tcbat [7]. The TcI and TcV DTUs are responsible for most human infections in Bolivia [8,9]. Crucially, the DTUs involved in creating antigens for commercially available tests may not correspond with those found in each country, potentially impacting these tests’ performance. Moreover, immune responses vary geographically, which could also impact a test’s ability to detect *T*. *cruzi* antibodies.

Given the low diagnosis rates of chronic *T*. *cruzi* infection, improvements in diagnostic policy and approaches are urgently needed. The present retrospective study was therefore designed to independently evaluate immunoassay tests for *T*. *cruzi* infection to generate unbiased performance data, under laboratory-controlled conditions. The index tests were commercially available LFAs, using the current CD diagnostic algorithm employed in Bolivia as the reference test method. The data generated, related to the optimal performances of the LFAs evaluated in this study, will help inform relevant stakeholders of these tests’ intended use and utility and assist in public health policymaking in relation to CD. The data will also be useful for informing national authorities about the potential use of LFAs in the detection of chronic cases of CD in regions with limited resources and in near-patient settings.

## Methods

### Ethics statement

All samples were obtained from patients as part of routine CD diagnosis with prior medical order. The Ethics Research Committee of the Faculty of Human Health Sciences at the Universidad Autonoma Gabriel Rene Moreno (Santa Cruz, Bolivia) approved the use in this study of the patient samples collected previously. All samples were collected from biorepositories of the participating regional reference laboratories and hospital laboratories, which are part of the National Program for the Surveillance and Control of Chagas of the General Directorate of Epidemiology, Ministry of Health and Sports of Bolivia. The sample collection has been retained for confirmation and further analyses of *T*. *cruzi* infections. Samples were assigned a code at the laboratory of origin, and no patient-identifiable information has been reported. All archived samples were de-identified to maintain participant confidentiality.

This retrospective, laboratory-based, diagnostic evaluation study sought to estimate the sensitivity/specificity of 10 commercially available LFAs for the serological diagnosis of chronic *T*. *cruzi* infection, using the current CD diagnostic algorithm employed in Bolivia as the reference test method. The hypothesis was that the diagnostic performance of the LFAs would be comparable to that of the serological laboratory-based tests used as reference standards in Bolivia. The study was performed at the Laboratory of Entomology and Parasitology, Instituto Nacional de Laboratorios de Salud (INLASA, La Paz, Bolivia).

### Participants

Samples were sourced from de-identified, remnant sera of patients collected as part of routine CD diagnosis in Bolivia in 2019, 2020, and 2021 and stored at the study site (INLASA). The de-identified samples had been transferred to the study site from the original laboratories, comprising six regional reference laboratories for CD and two hospital laboratories: Laboratorio Departamental De Referencia Sedes La Paz, Laboratorio Departamental De Referencia en Inmunologia Sedes Pando, Laboratorio Departamental De Referencia Sedes Chuquisaca, Laboratorio Departamental De Referencia en Inmunologia Sedes Beni, Laboratorio Departamental De Referencia Sedes Tarija, Laboratorio Departamental De Referencia Sedes Santa Cruz, Laboratorio De Analisis Clinico Estela (Oruro, Bolivia), Laboratorio Hospital Del Sud (Cochabamba, Bolivia).

At the study site, the subset of samples to be used in the study was selected from the collection according to the inclusion/exclusion criteria to obtain the minimum sample size, explained in detail below.

### Inclusion/exclusion criteria

The inclusion criteria were for remnant serum samples, stored at -20°C ±5°C, from individuals with suspected CD in Bolivia, with associated clinico-epidemiological data. Samples were excluded from the study if they had less than 1 ml of sample; they showed evidence of contamination; were poorly stored, labeled, or separated; exhibited hemolysis; had an unresolved serological classification; or lacked basic clinico-epidemiological data or routine diagnostic results for CD.

### Sample size

The sample size was determined to provide reasonable confidence and precision to estimate the performance of each test under evaluation in the detection of antibodies against *T*. *cruzi*. We based our calculations on sensitivity/specificity estimates ranging from 85% to 97.5%. A total of 151 positive and 151 negative samples would ensure an estimation of sensitivity and specificity, respectively, within the above range, with the following precision: 97.5% ± 2.5–95.0% ± 3.5–90.0% ± 4.8–85.0% ± 5.7, with an alpha error of 0.05 (95% confidence level) to describe performance (see S1 Table) [10,11]. Considering that around 30% of the specimens could have poor-quality, the minimum sample size required was estimated to be 200 positive and 200 negative samples.

### Sample selection

**Fig 1:**
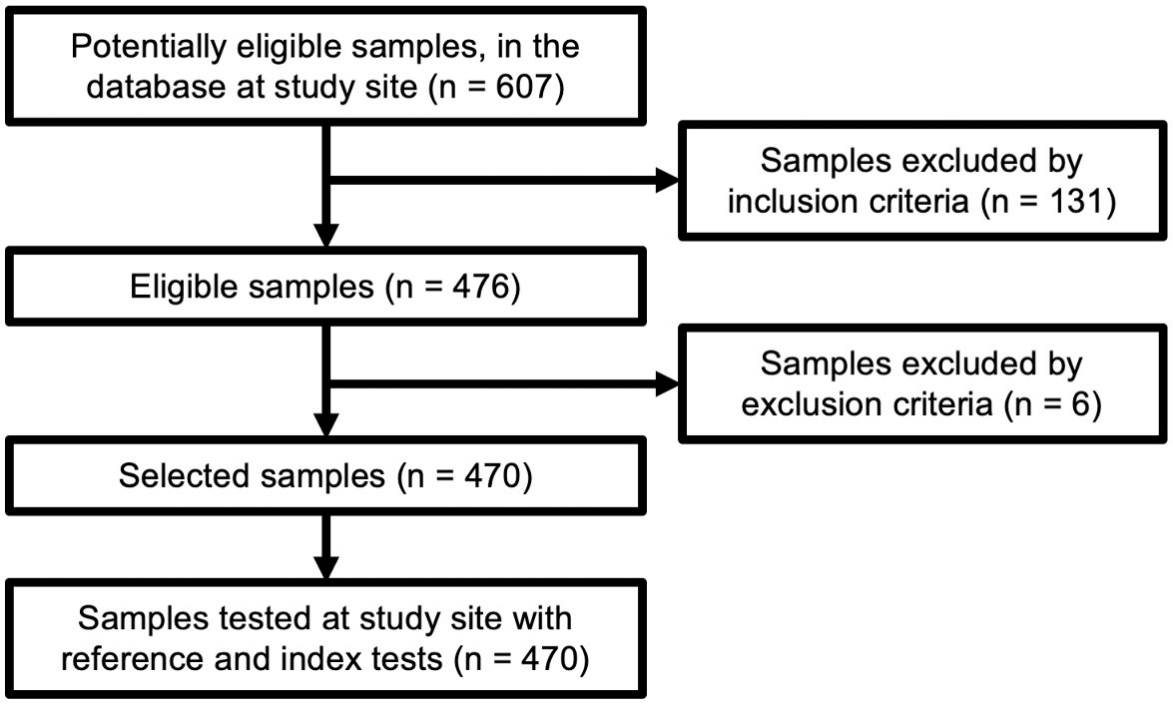
Sample selection flowchart.

Fig 1 shows the sample selection flowchart. A total of 607 samples were initially selected, by applying the inclusion criteria and using the database at the study site (INLASA). After applying the exclusion criteria, the collection of samples used in this study ultimately comprised 470 samples. Each sample was divided into seven aliquots; then, with each aliquot, either the reference tests were performed in parallel (two ELISAs plus HAI) or a maximum of four index tests were performed in parallel.

### Reference and index tests

At the study site, the samples were subjected to reference tests based on the current CD diagnostic algorithm and following the guidelines of the National Program for the Surveillance and Control of Chagas in Bolivia (*Programa Nacional de Vigilancia y Control de Chagas*, PNCCh) and the World Health Organization (WHO), i.e., two ELISA tests with different antigenic principles, in this case ELISA Chagas III (Grupo Bios, Chile) and Chagatest ELISA (Wiener Lab, Argentina). If the two ELISA results were conflicting, a third serological test, HAI, was performed. The reference test results obtained at the study site were considered for research-use only and were not used to diagnose patients with suspected CD.

The index tests included 10 commercially available LFAs that detect immunoglobulin G (IgG) antibodies specific to recombinant *T*. *cruzi* proteins (Table 1) and that could be procured from the manufacturer/local distributor at the time the study was developed. The LFAs were selected based on the following criteria: [1] LFA registered for use in Bolivia, [2] LFA used and registered in other CD-endemic countries, and [3] LFA produced in a CD-endemic country.

**Table 1:** The index tests (LFAs) used in this study (all used a cassette platform).

Test abbreviation	Assay name	Manufacturer (country)	Bolivian Sanitary Registry	Target; sensitivity (SE %) and specificity (SP %) declared by manufacturers in the instructions for use (country of origin of the samples / individuals tested)
ACCU	Accu Tell Chagas Ab Cassette	AccuBiotech CO LTD (China)	Yes	Target: not declared by the manufacturer; SE: 94.7, SP: 99.1 (unknown origin)
ACRO	Chagas Rapid Test Cassette WB/S/P	Acro Biotech (USA)	Yes	Target: not declared by the manufacturer; SE: 92.9, SP: 99 (unknown origin)
ARIA CTK	OnSite Chagas Ab Combo Rapid Test (Aria is the brand in Bolivia)	CTK Biotech (USA)	Yes	Target: recombinant *T*. *cruzi* antigens (not specified); SE: 92.9, SP: 100 (unknown origin)
ATLAS SENSO	*T*. *cruzi* IgG Chagas Test Cassette (Sensotest is the brand in Bolivia)	Atlas Link Technology (China)	Yes	Target: recombinant *T*. *cruzi* antigens (not specified); SE: 92.9, SP: 99.0 (unknown origin)
LEMOS	Chagas Rapido First Response	Laboratorios Lemos (Argentina)	Yes	Target: recombinant *T*. *cruzi* antigens (not specified); SE: >92.2, SP: >96 (study in Argentina)
SD-AB	SD Chagas Ab Rapid	Standard Diagnostic/Abbott (Korea)	Yes	Target: H49, 1F8; SE: 99.2, SP: 100 (unknown origin)
STATPAK	Chagas Stat-Pak Assay	Chembio (USA)	Yes	Target: B13, 1F8 and H49/JL7; SE: >98.5, >SP: 96 (studies in Brazil, Honduras, Venezuela, Bolivia, Argentina, El Salvador and Nicaragua)
TR-BIOM	TR Chagas—Biomanguinhos	Biomanginhos (Brazil)	No	Target: not declared by the manufacturer; SE: 99.4, SP: 98.5 (unknown origin)
WL	WL Check Chagas	Wiener Lab (Argentina)	Yes	Target: specific recombinant antigens from epimastigote and trypomastigote stages of *T*. *cruzi*; SE: >93.9, SP: >97.9 (unknown origin)
XERION	Xerion Chagas Ac Combo	Xerion (Colombia)	No	Target: recombinant *T*. *cruzi* antigens (not specified); SE: 92.9, SP: 100 (unknown origin)

Each index test was performed according to the manufacturer’s instructions for use (IFU). Each sample was tested once with each LFA index test, the results were interpreted by two independent operators at the study site, and the results were recorded independently. All test operators were blinded to the clinical characterization of the samples, *i*.*e*., *T*. *cruzi* infection positivity or negativity. A third staff member had the deciding vote for cases with discordant interpretations. Index test results (CD-positive or CD-negative) were based on agreement between the interpretation of at least two operators. The results of the study were used for research purposes only, not for the diagnosis of patients.

At the study site, each positive sample is routinely classified as having low, medium, or high reactivity, depending on the ELISA reference test antibody levels. For this study, we used the following subgroups for samples characterized as CD-positive at the study site, according to the IgG score with respect to the reference test value:

- Strongly positive: samples with high levels of reactivity in the serological reference tests
- Weakly positive: samples with low or medium levels of reactivity in the serological reference tests

### Data management and statistical analysis

Data generated during the study were first captured using paper forms and then entered into FIND’s online clinical trials platform, OpenClinica (Enterprise Edition version 4). Standardized, high-quality photographs of the index tests (LFAs) were taken using a smartphone-based app (TiraSpot, developed by SpotLab). The images collected were then coded. A serological external quality assurance panel (WHO International Standard 1st WHO anti-*Trypanosoma cruzi* I and II Antibody Reference Panel NIBSC code: 11/216) was used to corroborate the detection capacity of the 10 index tests (LFAs) with an international standard.

A statistical analysis plan was developed prior to the initiation of the study, and the parameters to be estimated included accuracy, sensitivity, specificity, balanced accuracy, agreement between each *T*. *cruzi* LFA and the current CD diagnostic algorithm, the stratified analysis, and the random forests algorithm to evaluate diagnostic performances in a combination setting (described in detail below). After the initial results were seen, we used the McNemar test to compare specificities and sensitivities across index tests and identify significant differences. Additionally, the failure rate of the LFAs, invalid test rate, were reported.

Sensitivity was defined as = [TP / (TP + FN)] x 100, where

- TP (true positive) was the number of positive index test results in agreement with *T*. *cruzi* infection positivity, and
- FN (false negative) was the number of negative index test results discordant with *T*. *cruzi* infection positivity

Specificity was defined as = [TN / (TN + FP)] x100, where

- TN (true negative) was the number of negative index test results in agreement with *T*. *cruzi* infection negativity, and
- FP (false positive) was the number of positive index test results discordant with *T*. *cruzi* infection negativity

The agreement was assessed through the calculation of Kappa coefficients (κ) [12], and the strength of agreement was interpreted as follows: poor (κ = 0), slight (0 < κ ≤ 0.20), fair (0.21 < κ ≤ 0.40), moderate (0.41 < κ ≤ 0.60), substantial (0.61 < κ ≤ 0.80), and almost perfect (0.81 < κ ≤ 1.0) agreement.

The two subgroups of CD-positive samples (based on the ELISA reference test antibody levels) were used for a stratified analysis of the primary endpoint (sensitivity of each index test), given that the estimated sample size necessary, a minimum of 51 samples per subgroup, was obtained to conduct this analysis.

### Ranking of the evaluated LFAs according to their relative importance in an LFA- combination setting to diagnose patients

We used a random forests algorithm [13] to evaluate diagnostic performances in a combination setting, using all available LFA results as input data. Of the samples, 70% were used for the training set, with the remaining 30% kept as a test set to report performances and the relative importance of each LFA (test´s impact on classification accuracy). A random forest model was trained and tested using the R package random forest function, *randomForest* (v4.7.1), using default parameters. For each LFA, we reported the mean decrease in the Gini index and used it as a ranking metric relative to the test´s impact on classification accuracy when included in a decision tree algorithm. The greater the decrease in the Gini index, the more important it is to rely on the given LFA when building the final decision tree.

### Test usability

The operational variables of the tests were compared, including the type of sample, sample volume required, reading time and temperature, storage temperature.

A usability score for each index test was established through the use of previously published standardized questionnaires [14]. These were completed by the test operators (highly skilled laboratory technicians at the study site), who provided their subjective assessment of the appearance of a test device’s background once the sample had been added, the intensity of the color of the control/test bands, the quality and comprehensiveness of the instructions for use, and the ease of interpreting the result. To measure the operators’ assessments, a value was assigned to each element in each category. Additionally, the usability score considered whether each index test included a sample dispenser in its commercial presentation. A usability score was obtained from the sum of all criteria, with a total possible score ranging from 5 to 12, with values greater than the average (8.5) representing the highest level of user-friendliness.

### Minimization of error and bias

Various steps were taken to minimize errors and the likelihood of bias. To prevent a prior diagnosis influencing the validity of the results, all tests, including the reference and index tests, were blinded at the study site and performed and analyzed independently. The sample collection comprised samples collected according to clearly defined eligibility criteria, and a random selection of the stored samples was used for the study. To avoid bias originating from a restricted participant population and *T*. *cruzi* lineages, the sample collection comprised samples from various geographic areas of Bolivia.

An external quality assessment (EQA) serological panel was obtained by the study site prior to commencing the study, as an external assessment of the laboratory’s ability to maintain satisfactory quality. This was also used with each different lot of LFA products. Results from reference tests were either generated automatically or were recorded blinded to the index test, eliminating the risk of review bias. To further reduce the overall risk of review bias, photographs of the LFAs evaluated were taken using a smartphone or a tablet, allowing standardized, high-quality photographs to be collected.

Samples for index test testing were the same samples that were used for reference testing, so disease progression bias was not a concern.

## Results

Of the 470 samples tested, the majority (359, 76.4%) were from females (Table 2). At the study site, using the reference tests, a total of 221 and 249 samples were confirmed as positive and negative, respectively, for chronic *T*. *cruzi* infection.

**Table 2:** Demographics, IgG anti-*T*. *cruzi* score, and location of residence of participants who provided samples used in this study.

Variable	Number	Percentage
**Age (years)**	**470**	**100%**
<18	21	4.5%
18–29	182	38.7%
30–39	119	25.3%
40–49	68	14.5%
50–60	46	9.8%
≥60	34	7.2%
Sex	**470**	**100%**
F	359	76.4%
M	111	23.6%
Pregnancy status	**327**	**100%**
No	157	35.8%
Yes	170	38.8%
IgG score	**470**	**100%**
Strongly positive	109	23.2%
Weakly positive	112	23.8%
Not applicable (negative)	249	53.0%
Department	**470**	**100%**
Beni	13	2.8%
Chuquisaca	94	20.0%
Cochabamba	60	12. 8%
La Paz	73	15.5%
Oruro	24	5.1%
Pando	45	9.6%
Potosí	30	6.4%
Santa Cruz	40	8.5%
Tarija	91	19.4%

The invalidity rate was estimated for each test during the study. The invalidity criteria were obtained from the IFU provided by the manufacturers and were related to the lack of a control band on a test. Four LFAs returned some results that were invalid (S2 Table); the remaining six LFAs returned no invalid results during the study.

### Clinical performance

**Fig 2:**
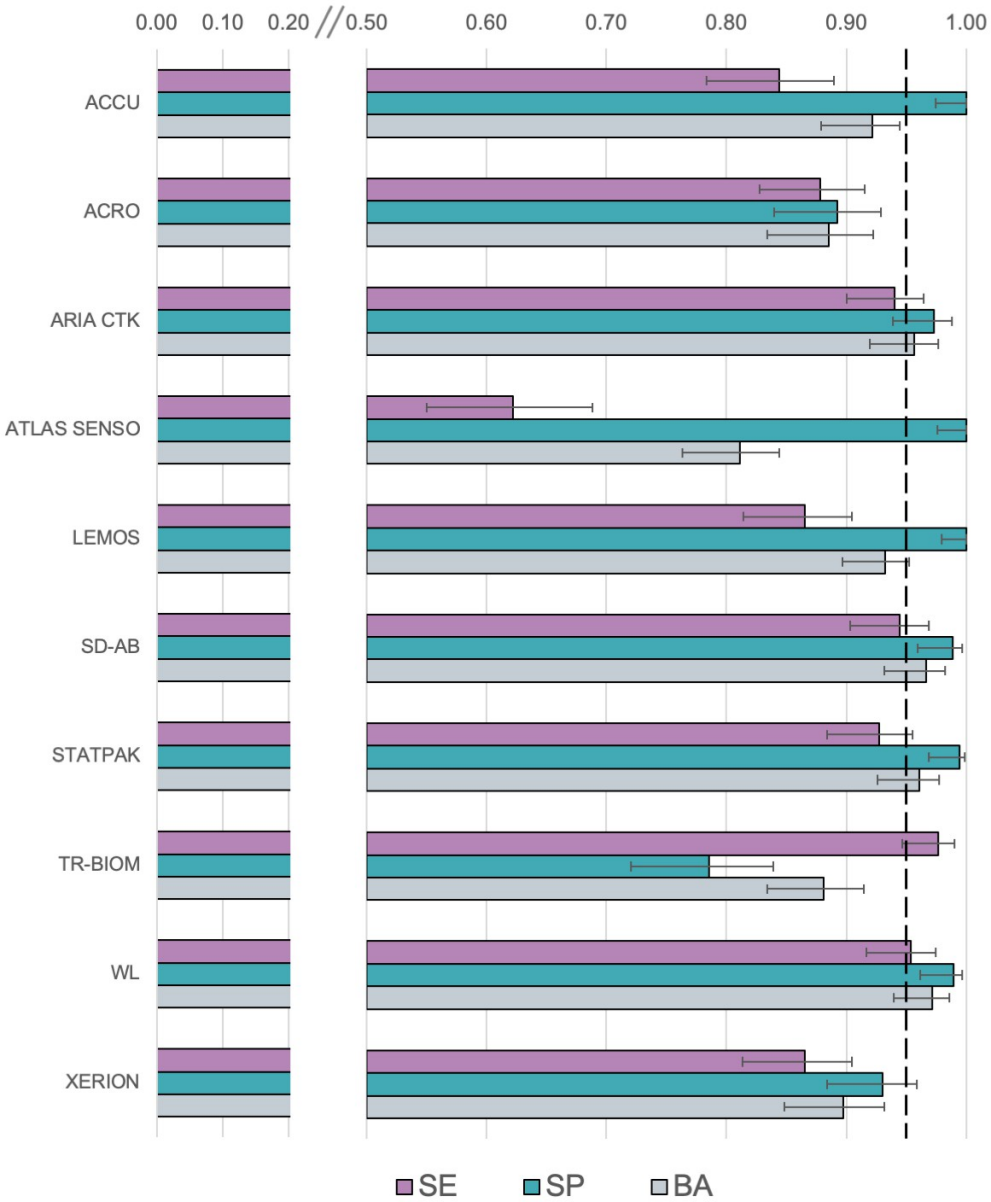
Sensitivity, specificity, and balanced accuracy for each index test (LFA); the dotted line indicates 95%.

Study samples were processed with the 10 index tests (LFAs) for detecting IgG anti-*T*. *cruzi* antibodies. Index test results considered the agreement between the interpretations of at least two test operators. Participants omitted from the analysis were i) participants with an interpretation from a single operator (186) or ii) participants with a discrepancy between two operators without the deciding vote of a third operator [17]. Table 3 shows the results obtained from the 10 LFAs evaluated, their clinical performance parameters, and the point estimates (%) and 95% confidence intervals (95% CI). Estimates of sensitivity, specificity, and balanced accuracy are shown in Fig 2.

**Table 3:** Results of the evaluated LFAs and their performance parameters.

Test	N	TP	FP	TN	FN	SE	SE [95% CI]	SP	SP [95% CI]	BA	BA [95% CI]	A	A [95% CI]	Kappa score
ACCU	328	151	0	149	28	84.36	[78.32–88.95]	100	[97.49–100.0]	92.18	[87.9–94.47]	91.46	[90–96%]	0.83
ACRO	400	188	20	166	26	87.85	[82.79–91.57]	89.25	[83.98–92.93]	88.55	[83.38–92.25]	88.5	[84–92%]	0.77
ARIA CTK	404	205	5	181	13	94.04	[90.07–96.48]	97.31	[93.86–98.85]	95.68	[91.97–97.67]	95.54	[93–98%]	0.91
ATLAS SENSO	344	115	0	159	70	62.16	[54.99–68.84]	100	[97.64–100.0]	81.08	[76.32–84.42]	79.65	[75–85%]	0.60
LEMOS	403	187	0	187	29	86.57	[81.38–90.49]	100	[97.99–100.0]	93.28	[89.68–95.25]	92.8	[90–96%]	0.86
SD-AB	375	187	2	175	11	94.44	[90.33–96.87]	98.87	[95.97–99.69]	96.66	[93.15–98.28]	96.53	[94–99%]	0.93
STATPAK	384	192	1	176	15	92.75	[88.39–95.56]	99.44	[96.87–99.9]	96.1	[92.63–97.73]	95.83	[93–98%]	0.92
TR-BIOM	398	211	39	143	5	97.69	[94.7–99.01]	78.57	[72.05–83.91]	88.13	[83.38–91.46]	88.94	[83–91%]	0.77
WL	403	206	2	185	10	95.37	[91.69–97.47]	98.93	[96.18–99.71]	97.15	[93.94–98.59]	97.02	[95–99%]	0.94
XERION	401	186	13	173	29	86.51	[81.3–90.44]	93.01	[88.41–95.87]	89.76	[84.85–93.16]	89.53	[84–92%]	0.79

A, accuracy; BA, balanced accuracy; FN, false-negative result (per LFA evaluated with respect to the reference test methods); FP, false-positive result (per LFA evaluated with respect to the reference test methods); N, number of samples (index test result with agreement of at least two operators); SE, sensitivity; SP, specificity; TN, true-negative result (per LFA evaluated with respect to the reference test methods); TP, true-positive result (per LFA evaluated with respect to the reference test methods).

Overall, inter-reader agreement computed over 3,827 reads was 97% (Kappa coefficient = 0.9).

The index test that obtained the highest agreement with the reference test was WL Check Chagas (Wiener Lab, Argentina), with a Kappa score of 0.9, while the index test that obtained the lowest agreement was the *T*. *cruzi* IgG Chagas Test Cassette—Sensotest (Atlas Link Technology, China), with a Kappa score of 0.6 (Table 3).

The WL Check Chagas (Wiener Lab, Argentina), SD Chagas Ab Rapid (Standard Diagnostic/Abbott, Korea), Chagas Stat-Pak Assay (Chembio, USA), and OnSite Chagas Ab Combo Rapid Test—Aria (CTK Biotech, USA) index tests achieved similar performances, with specificities of more than 95% while being more than 90% sensitive. Their sensitivities were all significantly higher when compared with the sensitivities of Xerion Chagas Ac Combo (Xerion, Colombia) (p < 0.026), Chagas Rapido First Response (Laboratorios Lemos, Argentina) (p < 1.5e-3), *T*. *cruzi* IgG Chagas Test Cassette—Sensotest (Atlas Link Technology, China) (p < 6.51e-12), and Accu Tell Chagas Ab Cassette (AccuBiotech CO LTD, China) (p < 3.28e-3) (S4–S7 Tables). Their specificities were all significantly higher than those of the Chagas Rapid Test Cassette WB/S/P (Acro Biotech, USA) (p < 2.25e-3) and TR Chagas—Biomanguinhos (Biomanguinhos, Brazil) (p < 8.64e-8). Most of the index test results that were discordant occurred in samples characterized as positive by the reference test method, i.e., most of the discordant results were false-negative results, except for TR Chagas—Biomanguinhos (Biomanguinhos, Brazil), which returned a higher proportion of false-positive results (Table 3).

The subgroups of CD-positive samples (i.e., strongly positive or weakly positive) were used to conduct a stratified analysis of the sensitivity of the evaluated LFAs (S3 Table).

In the case of the strongly positive subgroup, most of the LFAs evaluated (7/10) displayed sensitivities of more than 96% (>91% including their 95% CI lower bounds), except for *T*. *cruzi* IgG Chagas Test Cassette—Sensotest (Atlas Link Technology, China), Xerion Chagas Ac Combo (Xerion, Colombia), and Chagas Rapid Test Cassette WB/S/P (Acro Biotech, USA), which displayed sensitivities with 95% CI lower bounds of less than 90% (S3 Table). For the weakly positive subgroup, 9/10 LFAs displayed a point estimate sensitivity of between 47.6% and 90.6%, and only TR Chagas—Biomanguinhos (Biomanguinhos, Brazil) displayed a sensitivity of >95% (95%CI: 89.6–98.0%), having a significantly higher sensitivity than the other index tests (p < 0.041) except when compared with WL Check Chagas (Wiener Lab, Argentina) and SD Chagas Ab Rapid (Standard Diagnostic/Abbott, Korea), whose sensitivities still reached 90.6% (95%CI: 83.6–94.8%) and 89.6% (95%CI: 82.4–94.1%), respectively (S3 Table).

### Ranking of the evaluated LFAs according to their relative importance in an LFA- combination setting

We used a random forests algorithm to evaluate LFAs when used in combination to diagnose patients. The samples were split into training (70%) and test (30%) sets, and all 10 LFAs were used as input to the random forest model. The model achieved 96% accuracy with 93% sensitivity and 100% specificity on the test set. The LFAs were then ranked according to their relative importance in this random forest model, using the mean decrease in the Gini index as the ranking metric. The resulting ranking is shown in S1 Fig.

### Test usability

Qualitative data relating to the operating and storage requirements for each index test were obtained from the manufacturers’ IFU (S8 Table). The time required to perform the LFAs ranged from 15 to 35 minutes. All LFAs required the same operating temperature (15 to 30°C), had similar ranges of transport and storage temperatures (between 1 and 30°C), and similar in-use stability ranges (between 15 and 35 minutes after the addition of the buffer). For the sample type, all LFAs could be run with serum, plasma, or whole blood, with one exception (*T*. *cruzi* IgG Chagas Test Cassette—Sensotest, by Atlas Link Technology, China), which could only be run with serum or plasma. The whole-blood sample volume required ranged between 10 and 50 μl for all tests capable of running whole blood, except the SD Chagas Ab Rapid (Standard Diagnostic/Abbott, Korea), which required 100 μl.

A usability score for each test was estimated based on the subjective estimation of qualitative variables, related to the ease of use and interpretation of the results, by all laboratory technicians who participated in the study at the study site (S9 Table). These laboratory technicians assigned a usability score of more than average (>8.5) to most of the LFAs (8/10). In contrast, two LFAs received a usability score that was below average (7.0); therefore, according to the users, these tests were more difficult to handle or interpret. They were the Chagas Rapid Test Cassette WB/S/P (Acro Biotech, USA) and the *T*. *cruzi* IgG Chagas Test Cassette—Sensotest (Atlas Link Technology, China).

## Discussion

Diagnosis rates of chronic *T*. *cruzi* infection remain stubbornly low in countries where Chagas disease is endemic, including Bolivia. In seeking to improve the data available to inform public health policymaking in relation to CD, we conducted this study to independently evaluate LFAs for the detection of *T*. *cruzi* infection and generate unbiased performance data. The reference and index tests were performed by highly qualified laboratory staff using appropriately calibrated equipment and under carefully controlled conditions, including strict control of the storage temperature of samples and tests, processing temperature, and relative humidity.

Only four LFAs returned invalid results; these invalid results were considered to be an acceptable proportion, with less than 3% in each case.

Regarding the LFAs’ ability to detect truly infected patients (sensitivity), 4/10 LFAs had sensitivity point estimate values of more than 90%, and two of them had values of more than 95%. Regarding specificity, 6/10 LFAs showed a performance of more than 95% with their point estimates and confidence intervals. Most of the LFAs (6/10) evaluated showed performances that were comparable with current laboratory-based diagnostic methods in Bolivia (almost perfect agreement, with a Kappa score > 0.80).

Most of the incorrect results returned by LFAs corresponded to false-negatives, which could be attributable to the limit of detection (LOD). However, this parameter is not one that is specified by manufacturers. This phenomenon will need to be investigated further in future work.

The selection of the more challenging CD samples, classified as weakly positive samples with low or moderate reactivity, led to a decrease in the sensitivity values of the evaluated LFAs. However, these types of samples may not correspond to the use of LFAs in a real population. This part of the study was an attempt to assess the ability of the LFAs to detect infected individuals with low titers and to avoid the possible loss of infected individuals. When evaluating these samples in the stratified analysis, 1/10 LFAs displayed a sensitivity of more than 95% (TR Chagas—Biomanguinhos by Biomanguinhos, Brazil), followed by WL Check Chagas (Wiener Lab, Argentina) with 90.6% and SD Chagas Ab Rapid (Standard Diagnostic/Abbott, Korea) with 89.6%. However, TR Chagas—Biomanguinhos (Biomanguinhos, Brazil), is a test that does not have a sanitary registration in Bolivia (see Table 1) and displayed unbalanced proportions of performance and sacrificed specificity, with a higher proportion of false-positive results compared with WL Check Chagas (Wiener Lab, Argentina) and SD Chagas Ab Rapid (Standard Diagnostic/Abbott, Korea).

The four LFAs that showed the best individual performances, based on high values of sensitivity (93–95%) and specificity (97–99%), were WL Check Chagas (Wiener Lab, Argentina), SD Chagas Ab Rapid (Standard Diagnostic/Abbott, Korea), OnSite Chagas Ab Combo Rapid Test—Aria (CTK Biotech, USA), and Chagas Stat-Pak Assay (Chembio, USA). These tests also showed the highest relative importance and contribution if they were used in a combination setting for CD diagnosis, using a decision tree approach (sequential testing), and all have sanitary registrations in Bolivia. Interestingly, these tests also achieved the highest usability scores.

The LFAs evaluated had similar characteristics of usability. However, one LFA (SD Chagas Ab Rapid by Standard Diagnostic/Abbott, Korea) required a greater sample volume (100 μl) compared with the other LFAs (10–50 μl), while one LFA could not use whole blood as a sample type (*T*. *cruzi* IgG Chagas Test Cassette—Sensotest, by Atlas Link Technology, China). These attributes of these LFAs limit their usability in real-world conditions in point-of-care settings.

Evidence in the literature relating to the diagnostic performance of serological tests, including LFAs, in different settings is highly variable. Some authors have reported inconsistencies in serological test results due to parasite genetic diversity [15,16]; however, others have reported achieving similar results when using serological tests with sera from different countries and with lineages of pathogens from different endemic regions (DTUs TcI, II, and V) [17]. In the present study, we only used samples from Bolivian individuals for the test evaluation, being the same population to whom the test is intended to be applied, and where the TcI and TcV DTUs are responsible for most human infections [8]. Thus, for the LFAs evaluated here, additional studies in different CD-endemic populations will be required.

The overall lower sensitivities obtained in the present study, compared to the one declared by the test manufacturers in their IFUs (Table 1), could be related to the low affinity of circulating antibodies to the antigen test target, typical of endemic subpopulations. Whereas, we have used samples only from Bolivian individuals, and we did not select the samples based on the antibody levels, it is unclear whether the manufacturers used autochthonous subpopulations from Bolivia to carry out their diagnostic evaluations (only 3 manufacturers declare the origin of the subpopulations, coming mainly from Brazil and Argentina); and whether the manufacturers used only "strongly positive" and "strongly negative" samples without including those samples with low affinity, or those samples that present discordant results among the serological reference tests. Also, it is possible that the DTUs involved in creating the antigen test targets might not correspond to the prevalent DTUs in Bolivian human infections, resulting in a lower performance in this subpopulation from Bolivia. However, we cannot conclude whether it applies in this case, as only 2 manufacturers declare the target test antigens.

Thus, with a view of improving point-of-care testing for this neglected disease, and informing test developers and manufacturers about potential optimization needed of the available diagnostic tests, it might be pertinent that the regulatory authorities request that the manufacturers declare more detailed information regarding target test antigens, origin and selection of subpopulations used in their performance evaluations for granting the product registration.

Several limitations of our study should be noted. This was a retrospective study, and we did not assess safety, cost, LOD, or test kit repeatability. We used serum samples collected from a single country. While the LFAs that we used are also approved for use as point-of-care tests using (capillary) whole blood, we did not ascertain the clinical accuracy of these tests in the intended settings of use.

Commercially available LFAs for *T*. *cruzi* have begun to be used as screening tools for pregnant women in highly endemic areas with hard-to-reach populations where there are no high-level laboratory facilities in some CD-endemic countries, such as Bolivia. Our findings add to the increasing body of evidence being generated elsewhere [14,18,19] and suggest that LFAs are comparable to conventional serological tests. Other researchers have also indicated that LFAs could be used to confirm a diagnosis of *T*. *cruzi* chronic infection, if they were integrated with an algorithm [20,21]. This could help to increase access, at the point-of-care, to test-and-treat strategies for chronic CD in endemic countries.

Ultimately, achieving control on the way to the elimination of CD requires a radical simplification of the current CD testing pathways, to overcome current barriers to accessing CD treatment. Therefore, for the next stage of our work, field studies will be conducted to evaluate LFAs under real-world conditions, in point-of-care settings, and using whole blood as the sample type. These will be the available LFAs that showed the highest clinical performance under laboratory conditions in this study, but that also that comply with the criteria of affordability and appropriateness for low- and middle-income countries and point-of-care settings. Once the optimal performance is corroborated in field conditions, the LFAs could be incorporated into the diagnostic guidelines for chronic infection in Bolivia, thus helping improve health care coverage for people affected by Chagas disease.

## Supporting information

S1 Table Number of positive/negative samples needed for a range of estimated sensitivities/specificities. (DOCX)

S2 Table Number of invalid reads per LFA evaluated. (DOCX)

S3 Table Sensitivity of the evaluated LFAs for the antibody level subgroups (strongly and weakly positive). (DOCX)

S4 Table Significance of differences in sensitivity estimates between the LFAs evaluated (p-values of sensitivities in 2 by 2 comparisons) in the overall population. (DOCX)

S5 Table Significance of differences in specificity estimates between the LFAs evaluated (p-values of specificities in 2 by 2 comparisons) in the overall population. (DOCX)

S6 Table Significance of differences in sensitivity estimates between the LFAs evaluated (p-values of sensitivities in 2 by 2 comparisons) in the strongly-positive population subgroup. (DOCX)

S7 Table Significance of differences in sensitivity estimates between the LFAs evaluated (p-values of sensitivities in 2 by 2 comparisons) in the weakly-positive population subgroup. (DOCX)

S8 Table Operating and storage conditions of the LFAs evaluated. (DOCX)

S9 Table Usability scores of the evaluated LFAs. (DOCX)

S1 Fig Mean decrease in the Gini index of the 10 evaluated LFAs when used as input features to build a random forest model. (DOCX)

S1 Data Excel spreadsheet containing, in separate sheets, the underlying numerical data and statistical analysis for Figs 2 and S1. (XLSX)
