# Supplementary material for: Comparative evaluation of lateral flow assays to diagnose chronic Trypanosoma cruzi infection in Bolivia
Source: PLoS Negl Trop Dis. 2024 Mar 4;18(3):e0012016. doi: 10.1371/journal.pntd.0012016 (PMC10939271; doi:10.1371/journal.pntd.0012016)
Supplement: S1 Table — (DOCX) [file pntd.0012016.s001.docx]

**S1 Table. Number of positive/negative samples needed for a range of estimated sensitivities/specificities.**

| **Estimated sensitivity/specificity** | **95% confidence interval** | **Number of positive/negative samples** | **Number of positive/negative samples considering 30% poor-quality specimens** |
| --- | --- | --- | --- |
| 97.5% | ± 2.50% | ≥ 149.82 | 200 |
| 95.0% | ± 3.49% | ≥ 149.81 | 200 |
| 90.0% | ± 4.80% | ≥ 150.06 | 200 |
| 85.0% | ± 5.70% | ≥ 150.75 | 200 |
