# Supplementary material for: Comparative evaluation of lateral flow assays to diagnose chronic Trypanosoma cruzi infection in Bolivia
Source: PLoS Negl Trop Dis. 2024 Mar 4;18(3):e0012016. doi: 10.1371/journal.pntd.0012016 (PMC10939271; doi:10.1371/journal.pntd.0012016)
Supplement: S2 Table — (DOCX) [file pntd.0012016.s002.docx]

**S2 Table. Number of invalid reads per LFA evaluated.**

| Test | **Number of samples tested*** | **Number of invalid reads** | **Percentage of invalid reads** |
| --- | --- | --- | --- |
| ACRO | 400 | 11 | 2.8 |
| ATLAS SENSO | 344 | 1 | 0.3 |
| TR-BIOM | 398 | 2 | 0.5 |
| XERION | 401 | 8 | 2.0 |

*Index test result based on the agreement of at least two operators.
