# Supplementary material for: Comparative evaluation of lateral flow assays to diagnose chronic Trypanosoma cruzi infection in Bolivia
Source: PLoS Negl Trop Dis. 2024 Mar 4;18(3):e0012016. doi: 10.1371/journal.pntd.0012016 (PMC10939271; doi:10.1371/journal.pntd.0012016)
Supplement: S3 Table — (DOCX) [file pntd.0012016.s003.docx]

**S3 Table. Sensitivity of the evaluated LFAs for the antibody level subgroups (strongly and weakly positive).**

| **Test** | **N** | **TP** | **FN** | **SE** | **SE [95% CI]** | **N** | **TP** | **FN** | **SE** | **SE [95% CI]** |
| --- | --- | --- | --- | --- | --- | --- | --- | --- | --- | --- |
|  | **STRONGLY POSITIVE SAMPLES** | | | | | **WEAKLY POSITIVE SAMPLES** | | | | |
| **ACCU** | 99 | 96 | 3 | 96.97 | [91.47–98.96] | 80 | 55 | 25 | 68.75 | [57.93–77.85] |
| **ACRO** | 108 | 99 | 9 | 91.67 | [84.92–95.55] | 106 | 89 | 17 | 83.96 | [75.81–89.74] |
| **ARIA CTK** | 109 | 108 | 1 | 99.08 | [94.99–99.84] | 109 | 97 | 12 | 88.99 | [81.74–93.59] |
| **ATLAS SENSO** | 101 | 75 | 26 | 74.26 | [64.95–81.78] | 84 | 40 | 44 | 47.62 | [37.28–58.17] |
| **LEMOS** | 108 | 105 | 3 | 97.22 | [92.15–99.05] | 108 | 82 | 26 | 75.93 | [67.06–83.01] |
| **SD-AB** | 92 | 92 | 0 | 100 | [95.99–100.0] | 106 | 95 | 11 | 89.62 | [82.37–94.11] |
| **STATPAK** | 104 | 104 | 0 | 100 | [96.44–100.0] | 103 | 88 | 15 | 85.44 | [77.35–90.97] |
| **TR-BIOM** | 108 | 108 | 0 | 100 | [96.57–100.0] | 108 | 103 | 5 | 95.37 | [89.62–98.01] |
| **WL** | 109 | 109 | 0 | 100 | [96.6–100.0] | 107 | 97 | 10 | 90.65 | [83.65–94.84] |
| **XERION** | 108 | 95 | 13 | 87.96 | [80.49–92.83] | 107 | 91 | 16 | 85.05 | [77.08–90.58] |

CI, confidence interval; FN, false-negative; N, number of samples (index test result with the agreement of at least two operators); SE, sensitivity; TP, true positive.
