# Supplementary material for: Comparative evaluation of lateral flow assays to diagnose chronic Trypanosoma cruzi infection in Bolivia
Source: PLoS Negl Trop Dis. 2024 Mar 4;18(3):e0012016. doi: 10.1371/journal.pntd.0012016 (PMC10939271; doi:10.1371/journal.pntd.0012016)
Supplement: S4 Table — (DOCX) [file pntd.0012016.s004.docx]

**S4 Table. Significance of differences in sensitivity estimates between the LFAs evaluated (p-values of sensitivities in 2 by 2 comparisons) in the overall population.**

| **Test** | **ACRO** | **ACCU** | **ARIA CTK** | **ATLAS SENSO** | **LEMOS** | **XERION** | **SD AB** | **STATPAK** | **TR BIOM** |
| --- | --- | --- | --- | --- | --- | --- | --- | --- | --- |
| **ACCU** | 1,86E-01 |  |  |  |  |  |  |  |  |
| **ARIA CTK** | **1,23E-02** | **6,85E-04** |  |  |  |  |  |  |  |
| **ATLAS SENSO** | **6,77E-10** | **1,03E-07** | **3,09E-13** |  |  |  |  |  |  |
| **LEMOS** | 7,28E-01 | 5,05E-01 | **4,07E-04** | **4,87E-09** |  |  |  |  |  |
| **XERION** | 5,79E-01 | 7,28E-01 | **4,59E-03** | **2,97E-08** | 1,00E+00 |  |  |  |  |
| **SD-AB** | **1,39E-02** | **1,15E-03** | 1,00E+00 | **3,09E-13** | **1,77E-04** | **1,37E-03** |  |  |  |
| **STATPAK** | 6,62E-02 | **3,28E-03** | 5,46E-01 | **6,51E-12** | **1,50E-03** | **2,59E-02** | 1,31E-01 |  |  |
| **TR-BIOM** | **3,04E-05** | **7,56E-06** | **2,33E-02** | **3,41E-15** | **4,49E-06** | **6,46E-06** | 7,71E-02 | **9,37E-03** |  |
| **WL** | **3,51E-03** | **1,04E-04** | 6,83E-01 | **1,12E-13** | **1,04E-04** | **1,31E-03** | 1,00E+00 | 2,21E-01 | 1,31E-01 |
