# Supplementary material for: Comparative evaluation of lateral flow assays to diagnose chronic Trypanosoma cruzi infection in Bolivia
Source: PLoS Negl Trop Dis. 2024 Mar 4;18(3):e0012016. doi: 10.1371/journal.pntd.0012016 (PMC10939271; doi:10.1371/journal.pntd.0012016)
Supplement: S5 Table — (DOCX) [file pntd.0012016.s005.docx]

**S5 Table. Significance of differences in specificity estimates between the LFAs evaluated (p-values of specificities in 2 by 2 comparisons) in the overall population.**

| **Test** | **ACRO** | **ACCU** | **ARIA CTK** | **ATLAS SENSO** | **LEMOS** | **XERION** | **SD AB** | **STATPAK** | **TR BIOM** |
| --- | --- | --- | --- | --- | --- | --- | --- | --- | --- |
| **ACCU** | **6,15E-05** |  |  |  |  |  |  |  |  |
| **ARIA CTK** | **2,25E-03** | 7,36E-02 |  |  |  |  |  |  |  |
| **ATLAS SENSO** | **6,15E-05** | NA | 7,36E-02 |  |  |  |  |  |  |
| **LEMOS** | **2,15E-05** | NA | 7,36E-02 | NA |  |  |  |  |  |
| **XERION** | 7,71E-02 | **8,74E-04** | 6,14E-02 | **1,50E-03** | **8,74E-04** |  |  |  |  |
| **SD-AB** | **2,42E-04** | 4,80E-01 | 4,80E-01 | 4,80E-01 | 4,80E-01 | **9,37E-03** |  |  |  |
| **STATPAK** | **1,04E-04** | 1,00E+00 | 2,21E-01 | 1,00E+00 | 1,00E+00 | **2,57E-03** | 1,00E+00 |  |  |
| **TR-BIOM** | **6,05E-03** | **2,54E-08** | **8,64E-08** | **1,52E-08** | **1,17E-09** | **1,15E-04** | **9,08E-09** | **3,25E-09** |  |
| **WL** | **2,90E-04** | 1,00E+00 | 4,50E-01 | 1,00E+00 | 4,80E-01 | **9,82E-03** | 1,00E+00 | 1,00E+00 | **8,18E-09** |

NA: not applicable
