# Supplementary material for: Comparative evaluation of lateral flow assays to diagnose chronic Trypanosoma cruzi infection in Bolivia
Source: PLoS Negl Trop Dis. 2024 Mar 4;18(3):e0012016. doi: 10.1371/journal.pntd.0012016 (PMC10939271; doi:10.1371/journal.pntd.0012016)
Supplement: S6 Table — (DOCX) [file pntd.0012016.s006.docx]

**S6 Table. Significance of differences in sensitivity estimates between the LFAs evaluated (p-values of sensitivities in 2 by 2 comparisons) in the strongly-positive population subgroup.**

| **Test** | **ACRO** | **ACCU** | **ARIA CTK** | **ATLAS SENSO** | **LEMOS** | **XERION** | **SD AB** | **STATPAK** | **TR BIOM** |
| --- | --- | --- | --- | --- | --- | --- | --- | --- | --- |
| **ACCU** | 3,43E-01 |  |  |  |  |  |  |  |  |
| **ARIA CTK** | **2,69E-02** | 4,80E-01 |  |  |  |  |  |  |  |
| **ATLAS SENSO** | **8,56E-04** | **1,19E-04** | **3,86E-06** |  |  |  |  |  |  |
| **LEMOS** | 1,49E-01 | 1,00E+00 | 4,80E-01 | **4,40E-05** |  |  |  |  |  |
| **XERION** | 2,89E-01 | 6,14E-02 | **3,28E-03** | **1,19E-02** | **2,44E-02** |  |  |  |  |
| **SD-AB** | **2,33E-02** | 4,80E-01 | NA | **1,59E-06** | 4,80E-01 | **1,50E-03** |  |  |  |
| **STATPAK** | **7,66E-03** | 4,80E-01 | 1,00E+00 | **1,59E-06** | 4,80E-01 | **8,74E-04** | NA |  |  |
| **TR-BIOM** | **7,66E-03** | 2,48E-01 | 1,00E+00 | **9,44E-07** | 2,48E-01 | **8,74E-04** | NA | NA |  |
| **WL** | **7,66E-03** | 2,48E-01 | 1,00E+00 | **9,44E-07** | 2,48E-01 | **8,74E-04** | NA | NA | NA |

NA: not applicable
