# Supplementary material for: Comparative evaluation of lateral flow assays to diagnose chronic Trypanosoma cruzi infection in Bolivia
Source: PLoS Negl Trop Dis. 2024 Mar 4;18(3):e0012016. doi: 10.1371/journal.pntd.0012016 (PMC10939271; doi:10.1371/journal.pntd.0012016)
Supplement: S7 Table — (DOCX) [file pntd.0012016.s007.docx]

**S7 Table. Significance of differences in sensitivity estimates between the LFAs evaluated (p-values of sensitivities in 2 by 2 comparisons) in the weakly-positive population subgroup.**

| **Test** | **ACRO** | **ACCU** | **ARIA CTK** | **ATLAS SENSO** | **LEMOS** | **XERION** | **SD AB** | **STATPAK** | **TR BIOM** |
| --- | --- | --- | --- | --- | --- | --- | --- | --- | --- |
| **ACCU** | **9,52E-03** |  |  |  |  |  |  |  |  |
| **ARIA CTK** | 2,67E-01 | **1,95E-03** |  |  |  |  |  |  |  |
| **ATLAS SENSO** | **2,95E-07** | **5,20E-04** | **4,25E-08** |  |  |  |  |  |  |
| **LEMOS** | 8,09E-02 | 4,50E-01 | **1,15E-03** | **6,33E-05** |  |  |  |  |  |
| **XERION** | 1,00E+00 | **2,18E-02** | 4,23E-01 | **2,95E-07** | 8,09E-02 |  |  |  |  |
| **SD-AB** | 2,67E-01 | **3,28E-03** | 1,00E+00 | **1,06E-07** | **5,12E-04** | 2,67E-01 |  |  |  |
| **STATPAK** | 1,00E+00 | **9,37E-03** | 3,43E-01 | **2,31E-06** | **4,43E-03** | 1,00E+00 | 1,31E-01 |  |  |
| **TR-BIOM** | **3,28E-03** | **3,64E-05** | **4,12E-02** | **1,95E-09** | **2,15E-05** | **5,55E-03** | 7,71E-02 | **9,37E-03** |  |
| **WL** | 1,81E-01 | **5,12E-04** | 1,00E+00 | **6,34E-08** | **5,12E-04** | 3,02E-01 | 1,00E+00 | 2,21E-01 | 1,31E-01 |
