# Supplementary material for: Comparative evaluation of lateral flow assays to diagnose chronic Trypanosoma cruzi infection in Bolivia
Source: PLoS Negl Trop Dis. 2024 Mar 4;18(3):e0012016. doi: 10.1371/journal.pntd.0012016 (PMC10939271; doi:10.1371/journal.pntd.0012016)
Supplement: S8 Table — (DOCX) [file pntd.0012016.s008.docx]

**S8 Table. Operating and storage conditions of the LFAs evaluated.**

| **Test** | **Sample volume for WB** | **Assay time, excluding sample preparation** | **Sample type** | **Operating temperature** | **Transport and storage temperature** | **In-use stability** |
| --- | --- | --- | --- | --- | --- | --- |
| **ACCU** | 50 µl | 20 min | S, P, WB | 15–30°C | 2–30°C | Up to 15 min after addition of buffer |
| **ACRO** | 50 µl | 15 min | S, P, WB | 15–30°C | 2–30°C | Up to 20 min after addition of buffer |
| **ARIA CTK** | 40–50 µl | Up to 15 min | S, P, WB | 15–30°C | 2–30°C | Up to 15 min after addition of buffer |
| **ATLAS SENSO** | N/A | 15 min | S, P | 15–30°C | 2–30°C | Up to 20 min after addition of buffer |
| **LEMOS** | 10 µl | 20 min | S, P, WB | 15–30°C | 2–30°C | 20–30 min after addition of buffer |
| **SD-AB** | 100 µl | Up to 15 min | S, P, WB | 15–30°C | 1–30°C | Up to 15 min after addition of buffer |
| **STATPAK** | 10 µl | 15 min | S, P, WB | 18–30°C | 8–30°C | Up to 15 min after addition of buffer |
| **TR-BIOM** | 10 µl | 15 min | S, P, WB | 15–30°C | 2–30°C | Up to 20 min after addition of buffer |
| **WL** | 40 µl | 25–35 min | S, P, WB | 18–30°C | 2–30°C | Up to 35 min after addition of buffer |
| **XERION** | 50 µl | 15 min | S, P, WB | 15–30°C | 2–30°C | Up to 15 min after addition of buffer |

P, plasma; S, serum; WB, whole blood
