# Supplementary material for: Comparative evaluation of lateral flow assays to diagnose chronic Trypanosoma cruzi infection in Bolivia
Source: PLoS Negl Trop Dis. 2024 Mar 4;18(3):e0012016. doi: 10.1371/journal.pntd.0012016 (PMC10939271; doi:10.1371/journal.pntd.0012016)
Supplement: S9 Table — (DOCX) [file pntd.0012016.s009.docx]

**S9 Table.** **Usability scores of the evaluated LFAs**.

| **Test** | **Device background after testing** | **Test/control band intensity** | **Quality of IFU** | **Ease of interpreting the result** | **Sample dispenser included in the kit** | **Score** |
| --- | --- | --- | --- | --- | --- | --- |
|  | **Clear (2), dark (1)** | **Frequently intense (2),  frequently weak (1)** | **Very good (3),  good (2),  acceptable (1)** | **Effortless (3),  difficult (2),  very difficult (1)** | **Yes (2),  no (1)** |  |
| **ACCU** | 2.0 | 1.3 | 2.0 | 2.3 | 1.0 | **8.7** |
| **ACRO** | 1.3 | 1.0 | 1.7 | 2.0 | 1.0 | **7.0** |
| **ARIA CTK** | 2.0 | 2.0 | 2.0 | 2.7 | 2.0 | **10.7** |
| **ATLAS SENSO** | 1.3 | 1.0 | 1.7 | 2.0 | 1.0 | **7.0** |
| **LEMOS** | 1.3 | 1.3 | 2.0 | 2.3 | 2.0 | **9.0** |
| **SD-AB** | 2.0 | 1.7 | 2.3 | 3.0 | 1.0 | **10.0** |
| **STATPAK** | 2.0 | 1.3 | 2.0 | 3.0 | 2.0 | **10.3** |
| **TR-BIOM** | 2.0 | 1.0 | 1.3 | 2.0 | 2.0 | **8.3** |
| **WL** | 2.0 | 2.0 | 2.7 | 3.0 | 1.0 | **10.7** |
| **XERION** | 1.7 | 1.3 | 2.0 | 2.0 | 2.0 | **9.0** |
