# Supplementary material for: Comparative evaluation of lateral flow assays to diagnose chronic Trypanosoma cruzi infection in Bolivia
Source: PLoS Negl Trop Dis. 2024 Mar 4;18(3):e0012016. doi: 10.1371/journal.pntd.0012016 (PMC10939271; doi:10.1371/journal.pntd.0012016)

**S1 Fig.** **Mean decrease in the Gini index of the 10 evaluated LFAs when used as input features to build a random forest model.** This metric provides an estimation of each LFA’s relative importance and contribution if they were used in a combination setting for CD diagnosis, using a decision tree approach (sequential testing).


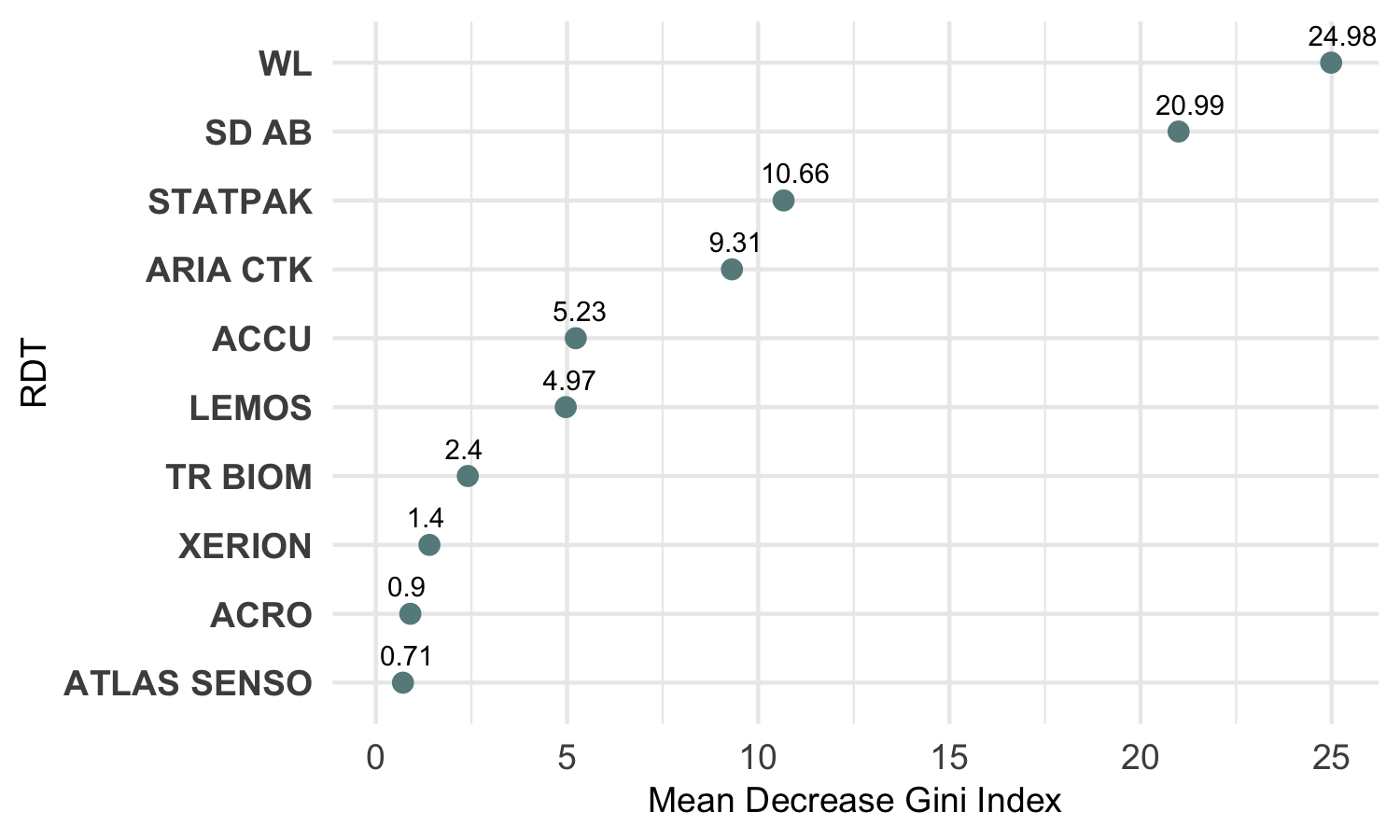

Supplement: S1 Fig — (DOCX) [file pntd.0012016.s010.docx]
